# DHAV-1 Inhibits Type I Interferon Signaling to Assist Viral Adaption by Increasing the Expression of SOCS3

**DOI:** 10.3389/fimmu.2019.00731

**Published:** 2019-04-09

**Authors:** Jinyan Xie, Mingshu Wang, Anchun Cheng, Xin-Xin Zhao, Mafeng Liu, Dekang Zhu, Shun Chen, Renyong Jia, Qiao Yang, Ying Wu, Shaqiu Zhang, Yunya Liu, Yanling Yu, Ling Zhang, Xiaoyue Chen

**Affiliations:** ^1^Institute of Preventive Veterinary Medicine, Sichuan Agricultural University, Chengdu, China; ^2^Key Laboratory of Animal Disease and Human Health of Sichuan Province, Sichuan Agricultural University, Chengdu, China; ^3^Avian Disease Research Center, College of Veterinary Medicine, Sichuan Agricultural University, Chengdu, China

**Keywords:** DHAV-1, IFNs, SOCS3, STATs, MX1

## Abstract

Duck hepatitis A virus type 1 (DHAV-1) is one of the most lethal pathogens in the duck industry. The attenuated vaccine (the CH60 strain) is cultivated through serial passage in chicken embryos and is widely used for the prevention and control of the disease. However, the specific mechanism underlying its adaptation in chicken embryos has not been fully elucidated. In this study, we first infected chicken embryo fibroblasts (CEFs) with the DHAV-1 CH60 strain. The peak of viral proliferation occurred within 36–48 h post-infection. The different DHAV-1 strains significantly induced the expression of IFNα, IFNγ, and Suppressor of cytokine signaling 3 (SOCS3) in CEFs, and we found that SOCS3 overexpression significantly promoted viral replication. Furthermore, SOCS3 overexpression significantly inhibited the expression of IFNα but promoted the expression of IFNγ. In addition, SOCS3 overexpression clearly decreased the mRNA levels of STAT1 and STAT3 in the Janus kinase (JAK)-STAT signaling pathway and inhibited the expression of the antiviral proteins MX1 and OASL. Immune-precipitation assays indicated that SOCS3 and IFNα do not physically interact. Subcellular localization of SOCS3 and IFNα revealed that SOCS3 was mainly located in the nucleus and cytoplasm, while IFNα was located only in the cytoplasm. Co-localization of these two proteins was not observed in the cytoplasm. In conclusion, the DHAV-1 CH60 strain may inhibit the expression of IFNα by increasing the SOCS3 protein and SOCS3 can in turn decrease STAT1 and STAT3 mRNA levels, thereby inhibiting the antiviral protein MX1 and ultimately promoting viral proliferation, indirectly assisting in viral adaptation in chicken embryos.

## Introduction

Duck hepatitis A virus type 1 (DHAV-1) is one of the most lethal pathogens for ducks, especially ducklings <1 week old, as it can cause 100% morbidity and 95% mortality (1). DHAV-1 is responsible for acute hepatitis characterized by petechial and ecchymotic hemorrhages of liver surfaces (2–5). To prevent and control the disease, we have established a variety of methods for virological detection of DHAV-1 and have characterized the functions of several viral proteins (6–11). At present, the prevention and control of the disease mainly depends on live attenuated vaccines (12, 13), such as the DHAV-1 CH60 strain (14). However, the focus and research on attenuated vaccines remain insufficient. The CH60 strain attenuated vaccine is cultivated through serial passage in chicken embryos and is widely used for the prevention and control of the disease. Intensive research has found that incompatible host translational selection pressure is one of the main mechanisms of viral attenuation and adaptation in chicken embryos (15). In addition, transcriptome sequencing has revealed that infection of chicken embryo livers with the CH60 strain is associated with enhanced type I and II interferon responses, activated innate immune responses, and abundant expression of cytokine signaling molecules 1 and 3 (SOCS1 and SOCS3) (16). Some structural and functional information about genes involved in host interactions with DHAV-1 has been reported recently (17–23). However, the specific mechanisms of viral attenuation and adaptation in chicken embryos remain to be further studied.

The SOCS/CIS family consists of CIS (cytokine-induced SH2-containing protein) and SOCS1 to SOCS7, each of which has a central SH2 domain, an amino-terminal domain of variable length and sequence, and a carboxy-terminal 40-amino-acid sequence known as the SOCS box (24). SOCS proteins regulate cytokine signaling mainly through the JAK-STAT signaling pathway, and SOCS1 and SOCS3 play an important role in the development of inflammation and tumors (25). The SOCS3 usually inhibits the activation of STAT3 via binding to both the JAK kinase and the cytokine receptor. Moreover, SOCS3 also plays a role in mediating the ubiquitination and subsequent proteasome degradation of cytokine/growth factor/hormone receptor (26). Previous studies have revealed that the CH60 strain significantly enhances SOCS1 and SOCS3 mRNA levels in the liver, activates the JAK-STAT signaling pathway and induces type I and II interferon responses (16). However, the role of SOCS proteins in viral attenuation and adaptation in chicken embryos remains unclear. Therefore, this study mainly explored whether the attenuated vaccine inhibits the JAK-STAT signaling pathway by overexpressing the SOCS3 protein, thereby inhibiting the expression of interferons and ultimately promoting its own replication to achieve adaptation.

## Materials and Methods

### Virus and Cells

The attenuated DHAV-1 CH60 strain and DHAV-1 WT strain were provided by the Instituted of Preventive Veterinary Medicine, Sichuan Agricultural University. Chicken embryo fibroblasts (CEFs) were cultured in minimum essential medium (MEM) supplemented with 10% newborn calf serum (Gibco) and incubated at 37°C with 5% CO_2_ in an incubator. The virus titer was measured as the 50% tissue culture infective dose (TCID_50_) according to previously described methods.

### RNA Isolation and cDNA Preparation

Total RNA was isolated using RNAiso Plus Reagent (TaKaRa, Dalian, China) according to the manufacturer's instructions. Genomic DNA was then removed and reverse transcription was performed using a PrimeScript™ RT Reagent Kit (Perfect Real Time, TaKaRa) according to the manufacturer's instructions.

### Viral RNA Load and Cytokine Expression in CEFs

The number of viral copies in total RNA was measured using methods previously established in our laboratory (7). Eight genes (IFNα, IFNβ, IFNγ, SOCS3, STAT1, STAT3, MX1, and OASL) and a housekeeping gene (β-actin) were analyzed by qPCR using primers designed with Primer Premier 5 (Table 1). The expression levels of immune-related genes were determined by qPCR using a SYBR® Premix Ex Taq™ II (Tli RNaseH Plus) Kit (TaKaRa) and an Applied CFX96 Real-Time PCR Detection System (Bio-Rad, Hercules, CA, USA). Amplification was performed in 10 μl reaction volumes containing 0.5 μl of each primer and 1 μl of cDNA. The following thermal cycling conditions were used: initial activation at 95°C for 30 s, 40 cycles of denaturation at 95°C for 5 s and annealing and extension at 58.6°C for 30 s, and a dissociation curve analysis step.

**Table 1:** Primers used in this study.

**Primer**	**Forward (5**′**-3**′**)**	**Reverse (5**′**-3**′**)**
gga-IFNα-qPCR	TCGCAACCTTCACCTCACC	CGCAGGCGCTGTAATCGT
gga-IFNβ-qPCR	TCCAGCTCCTTCAGAATACG	TGCGGTCAATCCAGTGTT
gga-IFNγ-qPCR	TCATACTGAGCCAGAvTTGT	AAGTCGTTCATCGGGAGC
gga-SOCS3-qPCR	CGGCACTTCTCACCCTCAG	CAGCTTCAGCACGCAGTCG
gga-STAT1-qPCR	GTAAAGAGGGAGCAATCA	TATCAGGGAAAGTAACAGC
gga-STAT3-qPCR	AAGCGTGGTCTCAGCATT	TGATTTGACAGCCCGAGTAG
gga-β-actin-qPCR	CACAGATCATGTTTGAGACCTT	CATCACAATACCAGTGGTACG
pCAGGS-IFNα-FLAG	CATCATTTTGGCAAAGAATTCACCGCCACCATGGCTGTGCCTGC AAGCCCA	TTGGCAGAGGGAAAAAGATCTCTACTTATCGTCGTCATCCTTGTAA TCAGTGCGCGTGTTGCCTG
pCAGGS-IFNβ-FLAG	CATCATTTTGGCAAAGAATTCACCGCCACCATGGCCACTGCAAAC CATCAGTC	TTGGCAGAGGGAAAAAGATCTTCACTTATCGTCGTCATCCTTGTA ATCCTGGGTGTTGAGACGTTT
pCAGGS-IFNγ-FLAG	CATCATTTTGGCAAAGAATTCACCGCCACCATGGCCACTTGCCAG ACTTACAA	TTGGCAGAGGGAAAAAGATCTTTACTTATCGTCGTCATCCTTGTA ATCGCAATTGCATCTCCTCT
pCAGGS-SOCS3-His	CATCATTTTGGCAAAGAATTCACCGCCACCATGGTCACCCACAG CAAGTT	TTGGCAGAGGGAAAAAGATCTTTAATGGTGATGGTGATGGTGGAG GGGGGCATCGTACT
DHAV-1 F1	CCTTAATTCAACGTCTAGCCCAC	TGCAAATCAGTTTCAAGGAGTTCT
DHAV-1 F2	CCCTATGCCATCTTGGATCT	CTTCCTGATTGAGTCCACAT
DHAV-1 F3	AATGTCCCAATACAAGGTGA	CCCCCAAAAATAAAATTTGAA
DHAV-1 F4	AGTCAGCATTAAATGGTGAAGT	CATATACCAAGAGGTTCAGGACG
DHAV-1 F5	CTTCAGTGGCTCCAGGA	TGATCTTTCCAAACCAACCA
DHAV-1 F6	CTTGGATTCTTGGTATAGGAAC	CCCATCACCATTCTATAAGC
DHAV-1 F7	ATGGCTAAGAAAGCATCT	GTAGGGTAGGGAATAGTAAAG

### Plasmids

To construct pCAGGS-IFNα-FLAG, pCAGGS-IFNβ-FLAG, pCAGGS-IFNγ-FLAG, and pCAGGS-SOCS3-His plasmids, IFNα (GenBank: NC_006127.5), IFNβ (GenBank: NC_006127), IFNγ (GenBank: NC_006088), and SOCS3 (GenBank: NC_006105.5) sequence were amplified from cDNA with PCR and primers (Table 1) and were integrated into the pCAGGS vector with a one-step cloning kit (Vazyme).

To compare the differential sequences of the DHAV-1 CH60 and DHAV-1 CH60 adapted CEF strains, we designed related primers according to the conserved sequence of DHAV-1 (F1–F7, Table 1). Amplification was performed in 30 μl reaction volumes containing 15 μl of PrimeSTAR Max DNA Polymerase (TaKaRa), 1.5 μl of each primer and 1 μl of cDNA. The following thermal cycling conditions were used: initial activation at 98°C for 3 min, 30 cycles of denaturation at 98°C for 10 s and annealing and extension at 55°C for 60 s. Fragment 1 and Fragment 7 were integrated into the pMD™19-T vector and then sequenced, and Fragment 2–6 were directly sequenced.

To prepare exogenous IFNα, CEFs were transfected with pCAGGS-IFNα-FLAG plasmids using Lipofectamine 3000 (Invitrogen) according to the manufacturer's instructions. At 48 h after transfection, cells were harvested and repeatedly frozen and thawed three times, then detected the concentration of IFNα by using chicken IFN-α ELISA kit (TW, shanghai). The concentration of recombinant IFNα was 100 pg/ml.

### Western Blot Analysis

CEFs were plated into 24-well cell culture dishes and cultured overnight. The cells were then transfected with the recombinant plasmids using Lipofectamine 3000 (Invitrogen) according to the manufacturer's instructions. At 36 and 48 h after transfection, cells were harvested and combined with 5 × sodium dodecyl sulfate (SDS) loading buffer and then resolved by SDS-polyacrylamide gel electrophoresis (PAGE). The proteins were then transferred onto polyvinylidene fluoride (PVDF) membranes. The membranes were blocked for 4 h with 5% non-fat milk/Tris-buffered saline with Tween-20 (TBST) at room temperature and then incubated with a primary antibody against FLAG (Proteintech Group, Wuhan, China), His (Abcam, Cambridge, UK) or STAT1 (CTS) for 12–24 h at 4°C. The membranes were rinsed (three times) with TBST and then incubated with a secondary antibody. The protein bands were detected using Western BLoT Chemiluminescence HRP Substrate (TaKaRa). Co-immunoprecipitation was performed as previously described (27).

### Indirect Immunofluorescence

Cells were transfected with the recombinant plasmids using Lipofectamine 3000 (Invitrogen) according to the manufacturer's instructions. At 48 h after transfection, the cells were rinsed (three times) with phosphate-buffered saline (PBS) and then fixed in 4% paraformaldehyde overnight. The cells were permeabilized (0.2% Triton X-100 for 25 min), incubated with blocking solution (5% bovine serum albumin (BSA) in PBS with Tween 20 (PBST) for 60 min at 37°C), incubated with a primary antibody against FLAG (Proteintech Group, Wuhan, China) or His (Abcam, Cambridge, UK) and then rinsed with PBST. The cells were then incubated with Texas red-conjugated goat anti-mouse IgG and fluorescein isothiocyanate (FITC)-conjugated goat anti-rabbit IgG secondary antibodies and finally treated with 4′,6-diamidino-2-phenylindole (DAPI). Images were captured using an 80i upright microscope (Nikon) and a SPOT Flex camera (28).

### shRNA-Mediated Knockdown of SOCS3

The pGPU6/GFP/Neo-SOCS3-275/371/663 expression vectors were designed and constructed by GenePharma (Shanghai, China), and the sequences of shRNAs were as follows: SOCS3-275 5′-GCTTCTACTGGAGCACGGTGA-3′, SOCS3-371 5′-GGCACTTCTTCACCCTCAGCG-3′, SOCS3-663 5′-GCACCTCTGCCGTAAGACTGT-3′. The CEFs were plated into 24-well cell culture dishes and cultured overnight. The cells were then transfected with the recombinant plasmids using Lipofectamine 3000 (Invitrogen) according to the manufacturer's instructions and infected with the 10^4^ TCID_50_ DHAV-1 CH60 adapted strain.

## Results

### Replication of DHAV-1 in CEFs

**Figure 1:**
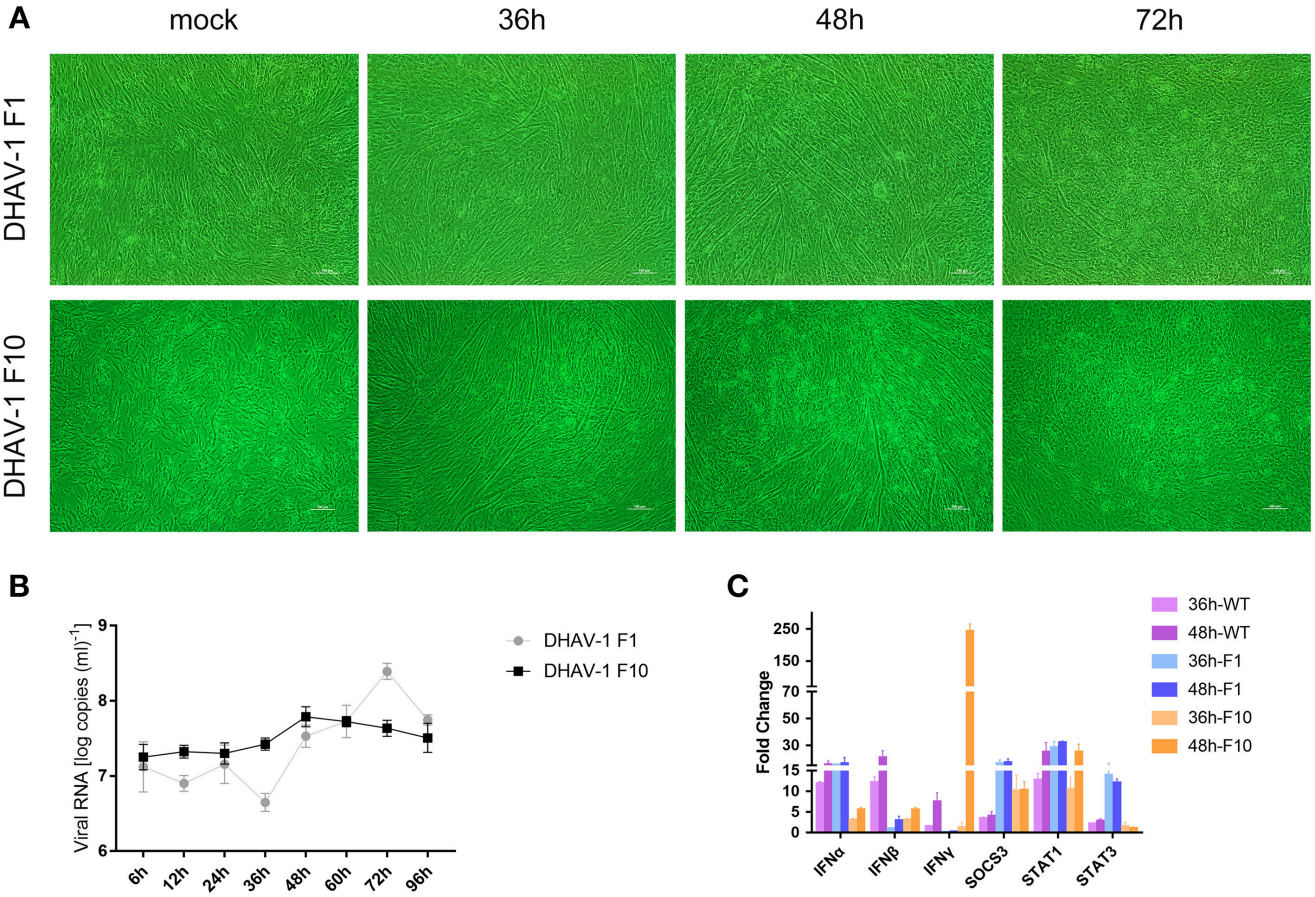
Dynamic changes in viral loads and immune-related genes in CEFs infected with the DHAV-1 CH60 strain. **(A)** Gross lesions in CEFs infected with the CH60 strain (F1) and adapted CH60 strain (F10). **(B)** Viral VP0 gene expression. The X-axis shows the different time points, and the Y-axis represents the logarithm of the number of viral RNA copies. **(C)** Cytokine expression levels of the CEF treated with DHAV-1 WT strain, CH60 strain (F1) and adapted CH60 strain (F10) were measured by the 2^−ΔΔ*Ct*^ method with relative quantification.

Previous studies have shown that chicken embryos can be used as an animal model for DHAV-1 (16). However, there is no report of DHAV-1 infection in CEFs. To further explore the interaction between DHAV-1 and the host immune system, the DHAV-1 CH60 strain was adapted to CEFs in 10 serial passages, and viral proliferation and lesions gradually stabilized in CEFs (Figure 1A). To further investigate the replication of DHAV-1 in CEFs, we infected CEFs with 10,000 TCID_50_ DHAV-1 and harvested cells to determine the viral copy number at 6, 12, 24, 36, 48, 60, 72, and 96 h post-infection (hpi). A significant proliferation of the DHAV-1 CH60 strain occurred from 36 to 72 hpi, a peak was observed at 72 hpi, and then a decreasing trend was observed at 96 hpi (Figure 1B, F1). The adapted strain (F10) presented more stable replication than the DHAV-1 CH60 strain, and the peak of proliferation (48 hpi) was earlier than that of the DHAV-1 CH60 strain (72 hpi). However, the peak copy number of the DHAV-1 CH60 strain was higher than that of the adapted strain. Compared with the DHAV-1 CH60 strain, the adapted strain had nine mutations, and the detailed sequence is shown in Supplementary Figure 3.. Previous studies have revealed that DHAV-1 CH60 strain infection significantly induces type I and II interferon responses, activates the JAK-STAT signaling pathway, and enhances SOCS3 mRNA levels in the livers of chicken embryos. We suspected that CH60 strain infection would also alter this pathway in CEFs. Therefore, we determined the mRNA levels of type I and II interferons, SOCS3 and STAT1/3 at 36 and 48 hpi. The three different DHAV-1 strains significantly induced the expression of IFNα, IFNβ, IFNγ, SOCS3, and STAT1 at 48 hpi. Notably, compared with the WT strain, the expression of type I IFN induced by the adapted strain (F10) decreased and the expression of SOCS3 increased (Figure 1C).

### Effects of IFNs and SOCS3 on DHAV-1 Replication

**Figure 2:**
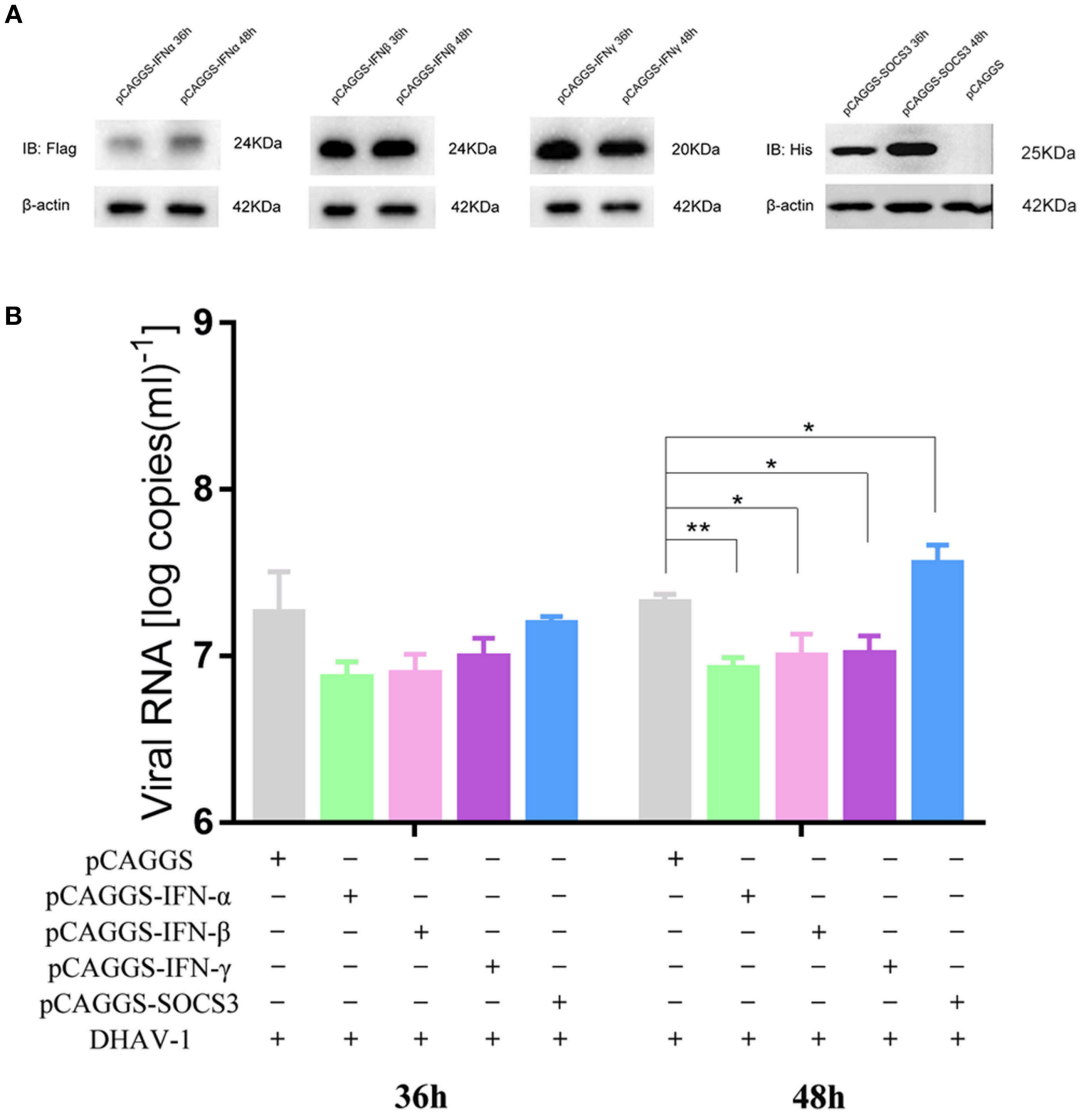
Effects of IFNs and SOCS3 on DHAV-1 replication. **(A)** Western blot analysis of IFNα/β/γ and SOCS3 expression. **(B)** CEFs were transfected with pCAGGS-IFNα-FLAG, pCAGGS-IFNβ-FLAG, pCAGGS-IFNγ-FLAG or pCAGGS-SOCS3-His and infected with 10,000 TCID_50_ of the DHAV-1 CH60 adapted strain. The viral copy number was determined at 36 and 48 hpi by quantitative PCR. Differences between two groups were analyzed using Student's *t*-test and considered significant at ^*^p < 0.05 and ^**^p < 0.01.

**Figure 3:**
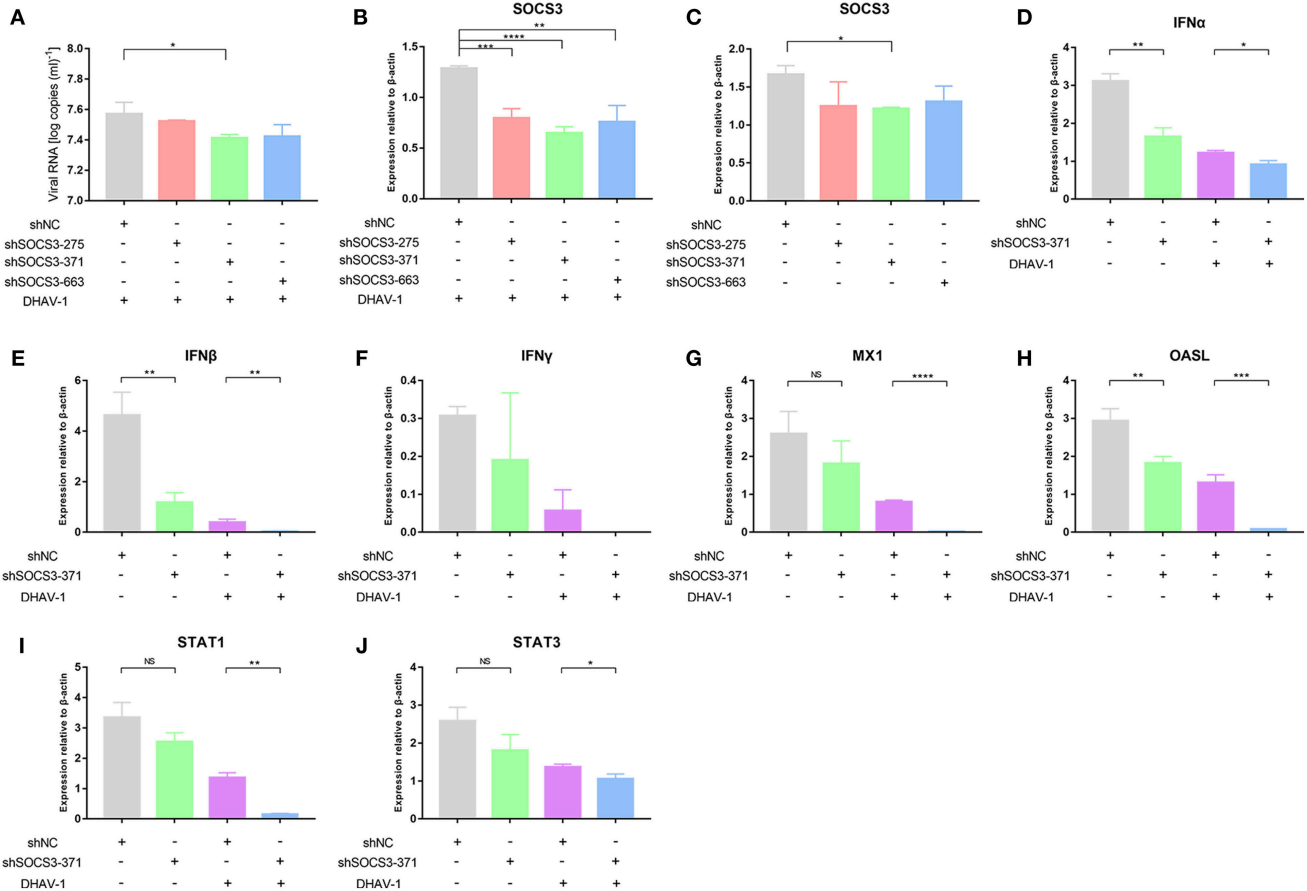
Effects of shSOCS3 on DHAV-1 replication and endogenous interferon pathway components. CEFs were transfected with pGPU6/GFP/Neo-SOCS3 and infected with 10^4^ TCID_50_ of the DHAV-1 CH60 adapted strain. The viral copy number was determined at 24 hpi by quantitative PCR. Quantitative PCR analysis of IFNα/β/γ, SOCS3, MX1, OASL, STAT1 and STAT3 mRNA levels. Differences between two groups were analyzed using Student's *t*-test and considered significant at ^*^p < 0.05, ^**^p < 0.01, ^***^p < 0.001, and ^****^p < 0.0001.

We demonstrated that DHAV-1 enhanced IFN and SOCS3 mRNA levels in CEFs. SOCS, a family of intracellular proteins, regulates the responses of immune cells to cytokines (29), while IFNs, as antiviral molecules, play a vital role in the immune system (30); however, the effects of IFNs and SOCS3 on the replication of DHAV-1 are still unknown. Therefore, we constructed four plasmids, including pCAGGS-IFNα-FLAG, pCAGGS-IFNβ-FLAG, pCAGGS-IFNγ-FLAG, and pCAGGS-SOCS3-His, that could successfully express in CEFs (Figure 2A). To further explore the effects of IFNs and SOCS3 on DHAV-1 replication, we transfected the above four plasmids into CEFs alone or in CEFs infected with 10,000 TCID_50_ of the DHAV-1 CH60 adapted strain and determined the viral copy number at 36 and 48 hpi by quantitative PCR. Compared with that in the empty vector group, DHAV-1 replication in the IFNα, IFNβ, and IFNγ groups was decreased at 36 h, although significant inhibition was not observed; in addition, SOCS3 did not show significant changes. IFNα, IFNβ, and IFNγ also significantly inhibited viral replication at 48 hpi, while SOCS3 significantly promoted viral replication (Figure 2B). Then, we designed three shRNAs against SOCS3 (shSOCS3-275/371/663) and found that shSOCS3-371 could significantly interfere with the transcription of SOCS3 (Figure 3C). We also found that viral replication decreased with the use of shSOCS3-371 (Figures 3A,B). Finally, the endogenous levels of the interferon pathway components (IFNα/β/γ, MX1, OASL, STAT1 and STAT3) were tested. Except for IFNγ, shSOCS3 could significantly inhibit the transcription of type I interferon, MX1, OASL, STAT1 and STAT3 (Figure 3).

### SOCS3 Inhibits the Expression of IFNα

**Figure 4:**
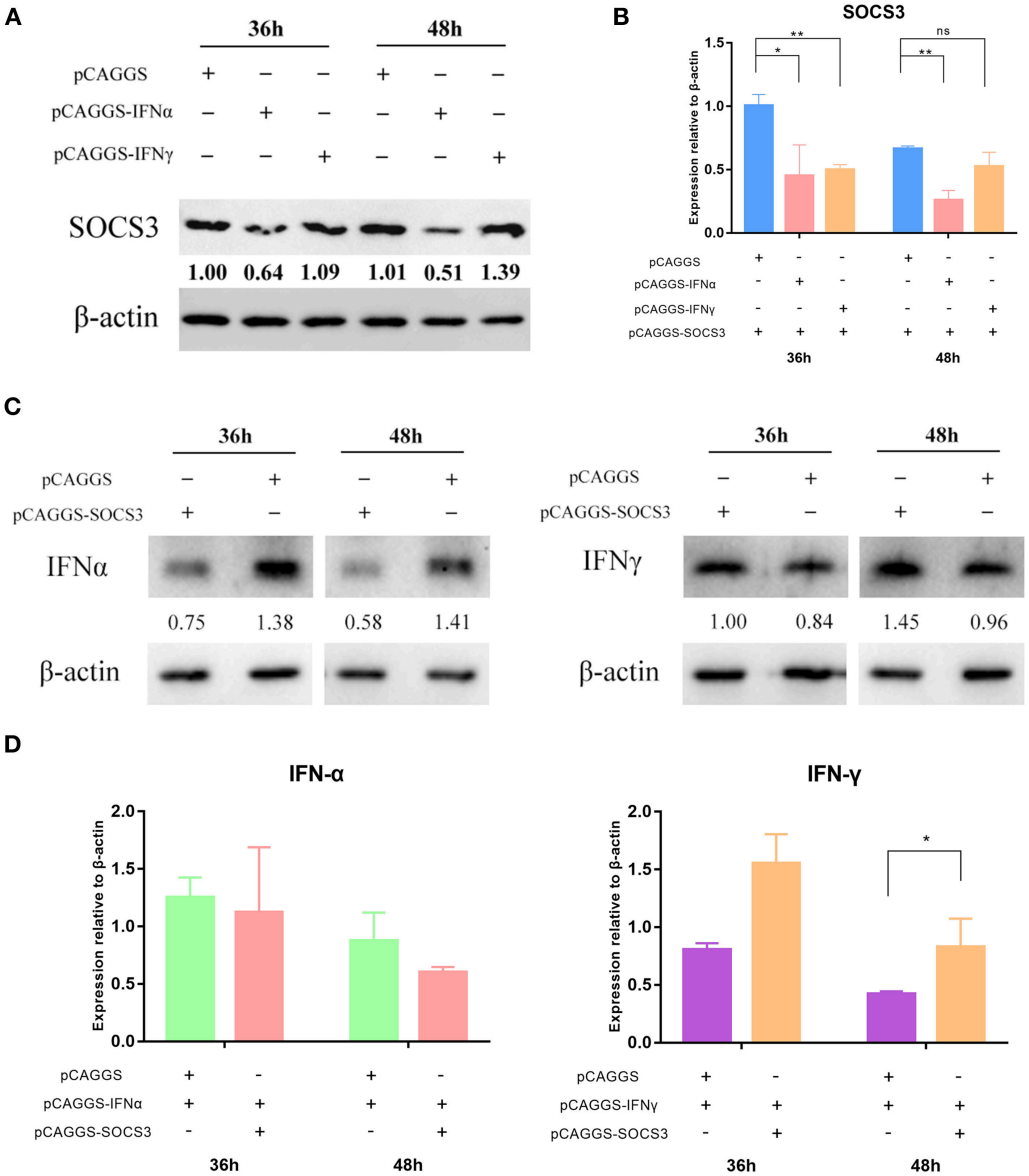
SOCS3 inhibits the expression of IFNα. pCAGGS-SOCS3-His and pCAGGS-IFNα/γ-FLAG were co-transfected into CEFs, which were harvested 36 and 48 h after transfection. **(A)** Western blot analysis of SOCS3 expression. **(B)** Quantitative PCR analysis of SOCS3 mRNA levels. **(C)** Western blot analysis of IFNα/γ expression. **(D)** Quantitative PCR analysis of IFNα/γ mRNA levels. Differences between two groups were analyzed using Student's *t*-test and considered significant at ^*^p < 0.05 and ^**^p < 0.01.

We suspected that the virus inhibited the expression of IFNs by hijacking the SOCS3 protein to promote viral replication. Therefore, to further explore how the virus used the SOCS3 protein to promote its own replication, we co-transfected pCAGGS-SOCS3-His and pCAGGS-IFNα-FLAG into CEFs and harvested the cells at 36 and 48 h after transfection. Western blot analysis was used to determine the expression of SOCS3 and IFNα. The results showed that IFNα significantly inhibited the expression of SOCS3 (Figure 4A; Supplementary Figure 1), while SOCS3 in turn also significantly inhibited the expression of IFNα (Figure 4C). In addition, SOCS3 and IFNα also inhibited each other at the transcriptional level (Figures 4B,D; Supplementary Figure 2). It is worth noting that we found no mutual inhibition between SOCS3 and IFNγ. In contrast, each of those proteins significantly promoted the expression of the other at 48 h after transfection (Figures 4A,C), and SOCS3 also significantly promoted IFNγ mRNA expression (Figure 4D).

**Figure 5:**
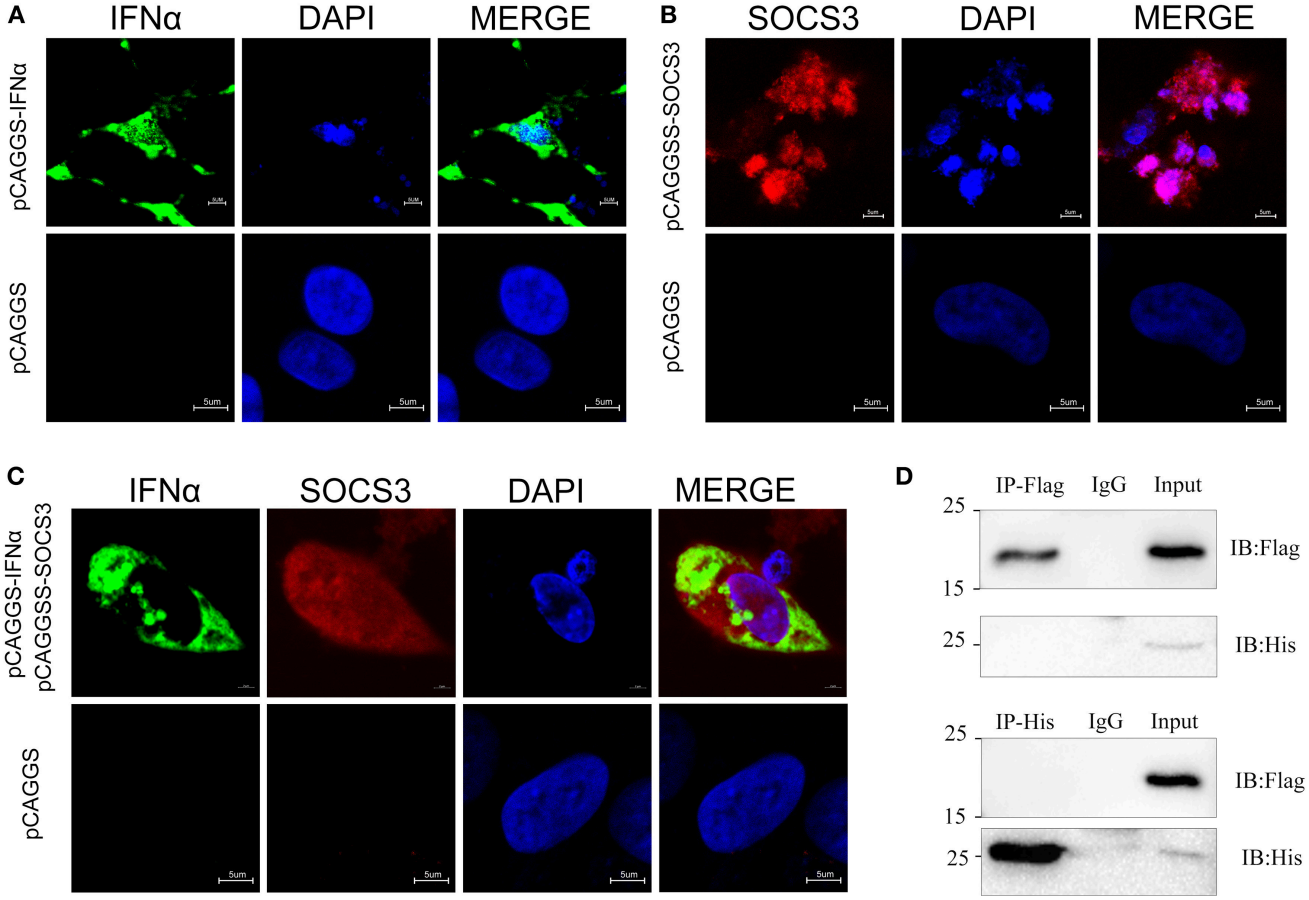
Subcellular localization of SOCS3 and IFNα. **(A)** Localization of IFNα (green) in CEFs. DAPI staining shows the nucleus in blue. **(B)** Localization of SOCS3 (red) in CEFs. DAPI staining shows the nucleus in blue. **(C)** Co-localization of IFNα (green) and SOCS3 (red) in CEFs. DAPI staining shows the nucleus in blue. **(D)** CEFs were transfected with pCAGGS-IFNα-Flag and pCAGGS-SOCS3-His for 48 h before the coimmunoprecipitation and immunoblot analysis with the indicated antibodies.

We confirmed that SOCS3 inhibited the expression of IFNα. To further explore the location of their interaction, we used indirect immunofluorescence to detect the subcellular localization of SOCS3 and IFNα. The results showed that IFNα is localized in the cytoplasm and is mainly located around the nucleus (Figure 5A), while SOCS3 is mostly localized in the nucleus, although some SOCS3 is also located around the nucleus (Figure 5B). In cells co-transfected with SOCS3 and IFNα, SOCS3 and IFNα were not co-localized in the cytoplasm (Figure 5C). Subsequently, we performed immune-precipitation assays and confirmed that IFNα and SOCS3 do not physically interact (Figure 5D; Supplementary Figure 4), suggesting that the interaction between these two proteins occurs indirectly in the cytoplasm.

### SOCS3 Inhibits the Expression of the Antiviral Proteins MX1 and OASL

**Figure 6:**
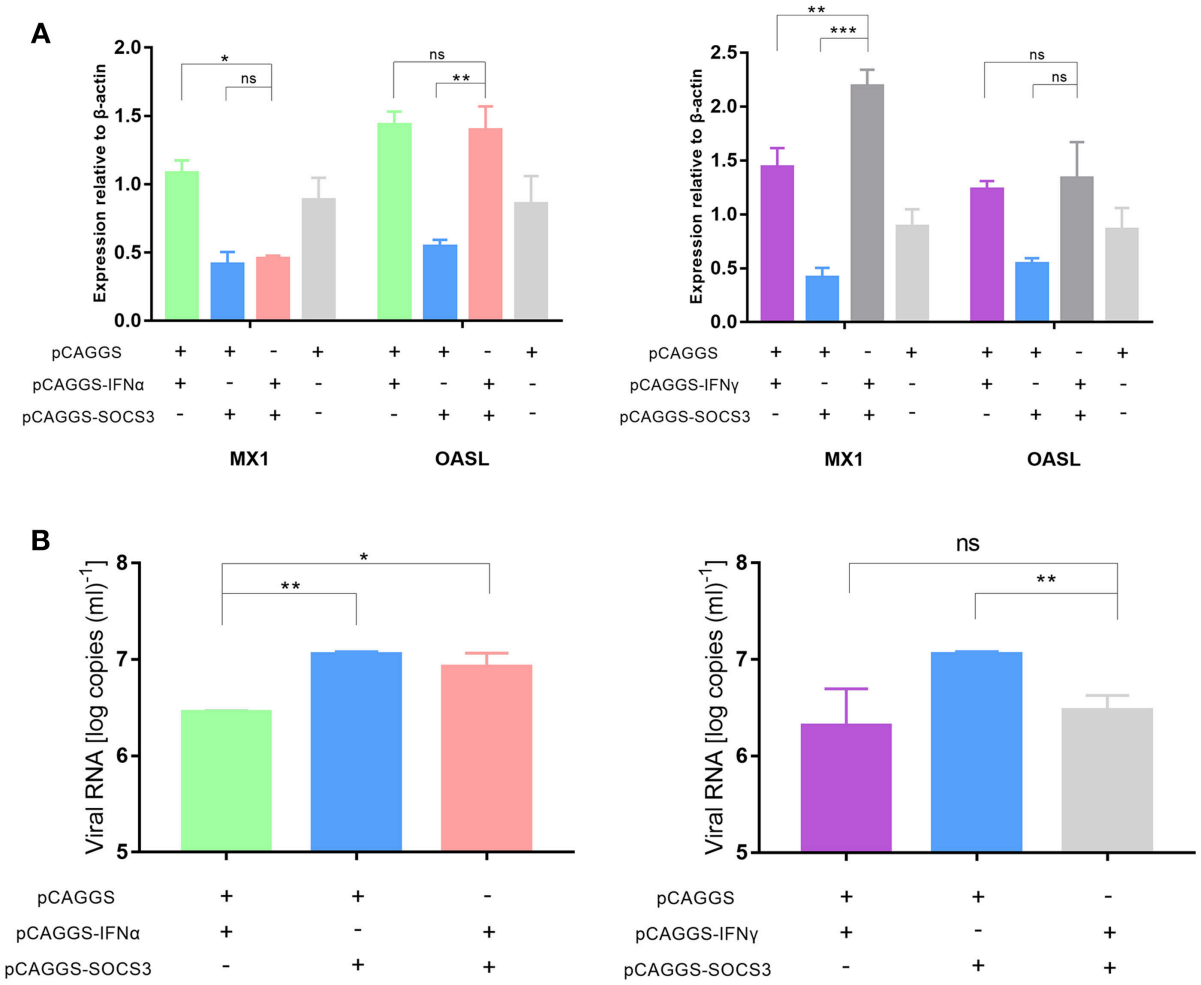
SOCS3 inhibits the expression of the antiviral proteins MX1 and OASL. pCAGGS-SOCS3-His and pCAGGS-IFNα/γ-FLAG were co-transfected into CEFs that were infected with 10,000 TCID_50_ of the DHAV-1 CH60 adapted strain. The cells were harvested 36 and 48 h after transfection. **(A)** Quantitative PCR analysis of the MX1 and OASL mRNA levels. **(B)** Quantitative PCR analysis of the viral copy number. Differences between two groups were analyzed using Student's *t*-test and considered significant at ^*^p < 0.05, ^**^p < 0.01, and ^***^p < 0.0001.

The innate immune system recognizes invasive viruses through pattern recognition receptors (PRRs) and then activates downstream pathways to produce IFNs; the IFNs then bind to corresponding receptors to activate the JAK-STAT signaling pathway, ultimately inducing antiviral responses to produce the antiviral proteins MX1, OASL, and others (31). Therefore, we wanted to further explore whether SOCS3 affected the expression of downstream antiviral proteins. Compared with that after IFNα and IFNγ transfection, the expression of MX1 and OASL was significantly inhibited after SOCS3 transfection. In addition, the expression of MX1, but not OASL, was significantly inhibited after co-transfection of IFNα and SOCS3 into CEFs compared with that after co-transfection of IFNα and pCAGGS (Figure 6A). These findings indicated that SOCS3 indeed inhibits the expression of IFNα and downstream MX1. Furthermore, we wanted to explore whether this process would ultimately promote viral replication. Therefore, we co-transfected SOCS3 and IFNα into CEFs and infected the cells with 10,000 TCID_50_ of the DHAV-1 adapted strain. The results showed that the viral copy number in the SOCS3 and IFNα-co-transfected group was slightly lower than that in the SOCS3 and pCAGGS-co-transfected group; however, the viral copy number was significantly upregulated compared with that in the IFNα and pCAGGS-co-transfected group (Figure 6B), indicating that SOCS3 inhibited the expression of MX1 and promoted viral replication. However, we found that SOCS3 did not inhibit the expression of IFNγ and thereby inhibited the expression of antiviral proteins, ultimately affecting viral replication.

### SOCS3 Inhibits the Expression of STAT1 and STAT3

**Figure 7:**
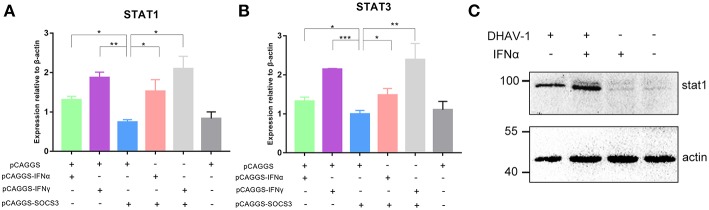
SOCS3 inhibits the expression of STAT1 and STAT3. **(A,B)** pCAGGS-SOCS3-His and pCAGGS-IFNα/γ-FLAG were co-transfected into CEFs that were infected with 10,000 TCID_50_ of the DHAV-1 CH60 strain. The cells were harvested 36 and 48 h after transfection. Quantitative PCR was used to analyse the STAT1 and STAT3 mRNA levels. Differences between two groups were analyzed using Student's *t*-test and considered significant at ^*^p < 0.05, ^**^p < 0.01, and ^***^p < 0.0001. **(C)** Western blot analysis of STAT1 expression. DHAV-1 infected CEFs at 48 hpi and after the addition of 100 pg IFNα to stimulate CEFs at 37°C for 15 min. Cells were harvested after stimulation.

The JAK-STAT signaling pathway plays an important role in the induction of host cytokines. To further explore whether SOCS3 inhibited the production of antiviral molecules by affecting the JAK-STAT signaling pathway, we determined the transcript levels of STAT1 and STAT3 and found that SOCS3 significantly inhibited the expression of STAT1 and STAT3 compared to IFNα/γ, suggesting that SOCS3 can indeed affect the JAK-STAT signaling pathway. However, when IFNα or IFNγ was co-transfected with SOCS3, STAT1 and STAT3 were not significantly inhibited, indicating that SOCS3 could not inhibit the expression of STAT1 and STAT3 by directly inhibiting IFNα (Figures 7A,B). Then, we detected the expression of STAT1 and found that DHAV-1 could induce the expression of STAT1, with increased STAT1 levels observed in the infected cells in the presence of IFNα (Figure 7C).

## Discussion

**Figure 8:**
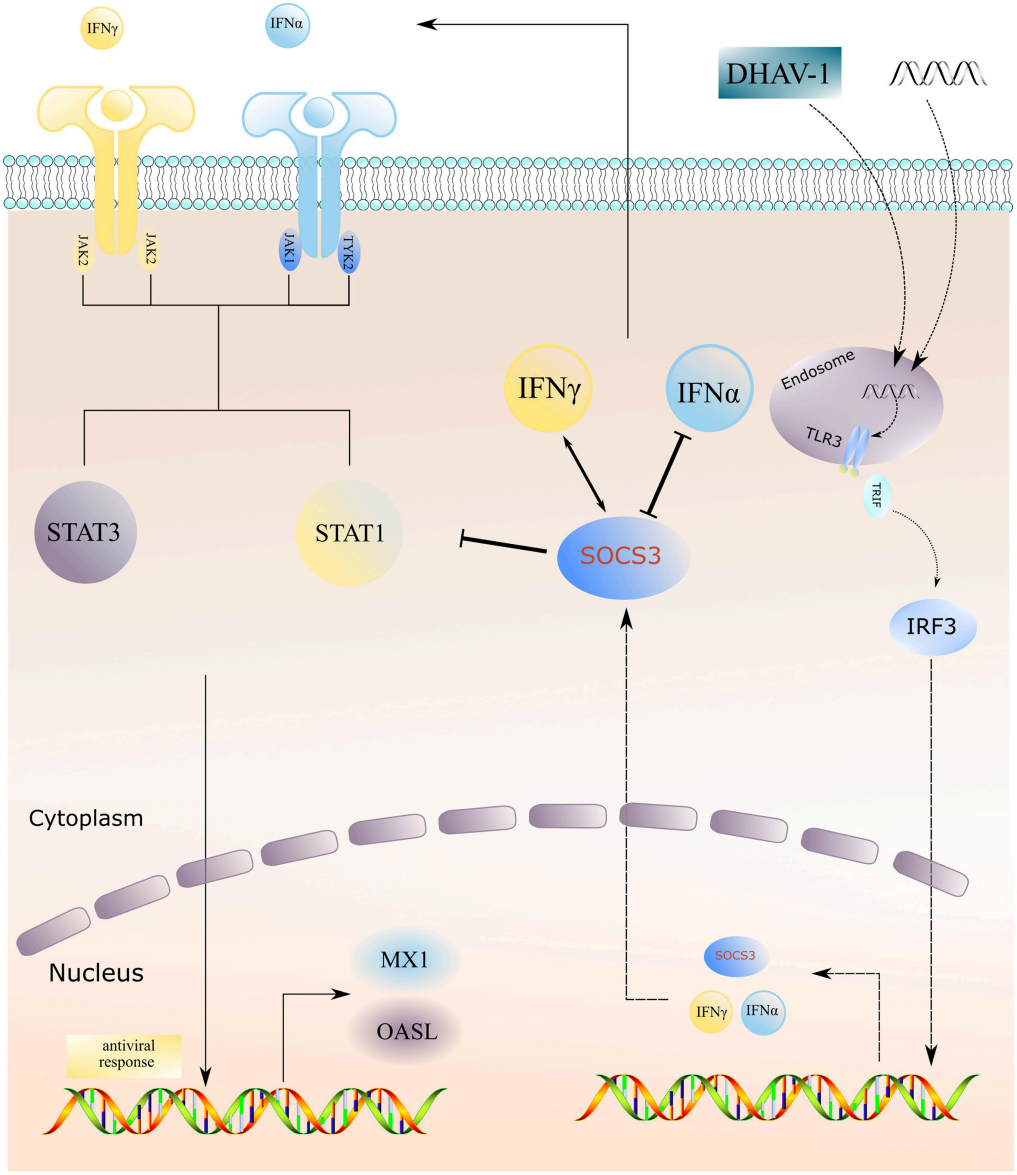
Schematic model of DHAV-1 inhibiting type I interferon signaling by hijacking the SOCS3 protein. Overexpression of the SOCS3 protein inhibits the expression of IFNα; in addition, SOCS3 inhibits the expression of the key molecules STAT1 and STAT3 in the downstream JAK-STAT pathway and inhibits the expression of the terminal antiviral protein MX1, ultimately promoting virus replication and indirectly assisting in viral adaptation.

Previous studies have shown that the CH60 strain attenuated vaccine undergoes different types of mutations during serial passage, such as synonymous and non-synonymous mutation in the coding region and mutation in the UTRs. Conceivably, these mutations drive the adaptation of the virus to the environment (15). The changes in attenuated vaccines and virulent strains in the host before and after adaptation are also worthy of attention. Through transcriptome sequencing, we have found that attenuated vaccines induce type I and II interferon responses, activate innate immune responses, and simultaneously enhance the expression of SOCS1 and SOCS3 (16). We suspected that the adaptation of attenuated vaccines during the process of serial passage is related to high expression of SOCS proteins. Further investigation revealed that overexpression of the SOCS3 protein inhibited the expression of IFNα; in addition, SOCS3 inhibited the expression of the key molecules STAT1 and STAT3 in the downstream JAK-STAT pathway and inhibited the expression of the terminal antiviral protein MX1, ultimately promoting CH60 strain replication and thus indirectly assisting in viral adaptation (Figure 8).

Innate immunity is the first line of host defense against invading pathogens (32). In innate immunity, invading pathogens are recognized by PRRs, stimulating the downstream signaling pathway to produce type I interferons, which then activate the JAK-STAT signaling pathway; this process ultimately induces the expression of IFN-stimulated genes (ISGs) and leads to the destruction of the pathogens. However, to replicate and survive, attenuated viruses have evolved a complete immune evasion mechanism to achieve their own replication (33). The induction of the SOCS3 protein by attenuated vaccines has drawn our attention, and we have further confirmed that SOCS3 can significantly inhibit the expression of IFNα and the antiviral protein MX1 to promote viral replication, which is consistent with our hypothesis. Another study on HCV similarly found that HCV evades immune responses by hijacking SOCS function; the HCV core protein induces SOCS3 expression and the inhibition of STAT1 activation, tyrosine phosphorylation and nuclear localization, ultimately neutralizing the antiviral effects of IFNs (34). Moreover, the data indicate that higher hepatic SOCS3 expression is associated with a poor response to antiviral therapy in human HCV patients (35). However, we found that SOCS3 could not inhibit type II interferon or IFNγ but rather had a promoting effect. IFNγ bridges innate and adaptive immunity by modulating the differentiation of naive T cells into either Th1 (cell immunity) or Th2 (humoral immunity) cells, while it directs long-term control of viral infection by inducing cytotoxic immunity through the recognition of cell surface-bound viral antigens complexed with MHC proteins (30). In addition, it has been demonstrated that SOCS1 is a potent inhibitor of the IFNγ signaling pathway (36, 37) and can prevent atherosclerosis by inhibiting IFNγ signaling (38). Therefore, we hypothesize that attenuated vaccines may regulate IFNγ signaling through SOCS1 rather than SOCS3, and the specific mechanism needs further investigation.

The JAK-STAT signaling pathway manages more than 50 downstream cytokines and growth factors and is regarded as a central communication node for the immune system. This pathway is mainly composed of Janus kinases (JAKs) and signal transducers and activators of transcription (STATs); JAKs can be subdivided into JAK1, JAK2, JAK3, and TYK2, while STATs can be subdivided into STAT1, STAT2, STAT3, STAT4, STAT5A, STAT5B, and STAT6 (39). A variety of viruses disrupt the IFNα signaling pathway by degrading the major components of the JAK-STAT signaling pathway. For example, the HIV Vif protein achieves immune evasion by disrupting the IFNα-mediated phosphorylation of STAT1 and STAT3 and reducing the expression of ISG15 (31). However, enterovirus 71 (EV71) inhibits the intracellular type I IFN signaling pathway by downregulating the expression of the JAK1 protein (40). We found that overexpression of SOCS3 significantly reduced the transcript levels of STAT1 and STAT3 and the antiviral proteins MX1 and OASL. However, when SOCS3 was co-transfected into cells with IFNα, we found that the mRNA levels of STAT1 and STAT3 were not significantly reduced. We hypothesize that SOCS3 may inhibit the phosphorylation of STAT1 or STAT3, thereby affecting the expression of downstream MX1 and ultimately destroying the type I IFN signaling pathway.

In summary, the DHAV-1 CH60 strain inhibits the expression of IFNα by increasing the SOCS3 protein and SOCS3 then inhibits the mRNA expression of STAT1, STAT3 and the antiviral protein MX1, which ultimately promotes viral replication and thus indirectly assists in viral adaptation.

## Ethics Statement

The study was approved by the Committee of Experiment Operational Guidelines and Animal Welfare of Sichuan Agricultural University (the approved permit number is XF2014-18). Experiments were conducted in accordance with approved guidelines.

## Author Contributions

JX conceived and carried out the experiments, analyzed the data, and wrote the manuscript. AC and MW conceived and supervised the study. X-XZ, ML, DZ, SC, RJ, YW, SZ, YL, YY, LZ, XC, and QY interpreted the data and revised the manuscript. All the authors reviewed the manuscript.

### Conflict of Interest Statement

The authors declare that the research was conducted in the absence of any commercial or financial relationships that could be construed as a potential conflict of interest.
