# Supplementary material for: DHAV-1 Inhibits Type I Interferon Signaling to Assist Viral Adaption by Increasing the Expression of SOCS3
Source: Front Immunol. 2019 Apr 9;10:731. doi: 10.3389/fimmu.2019.00731 (PMC6465609; doi:10.3389/fimmu.2019.00731)
Supplement: Supplementary file 1 [file Data_Sheet_1.docx]

***Supplementary Material***

**DHAV-1 inhibits type I interferon signalling to assist viral adaption by increasing the expression of SOCS3**

**Jinyan Xie^1,2^****^¶^, Mingshu Wang^1,2,3¶^, Anchun Cheng^1,2,3*^,** **Xin-Xin Zhao^1,2,3^, Mafeng Liu^1,2,3^, Dekang Zhu^2,3^, Shun Chen^1,2,3^,** **Renyong Jia^1,2,3^,** **Qiao Yang^1,2,3^,** **Ying Wu^1,2,3^,** **Shaqiu Zhang^1,2,^****^3^, Yunya Liu^1,2,3^,** **Yanling Yu^1,2,3^,** **Ling Zhang^1,2,3^,** **Xiaoyue Chen^1,2,3^**

Corresponding authors: Anchun Cheng, chenganchun@vip.163.com

**Supplementary Figures**


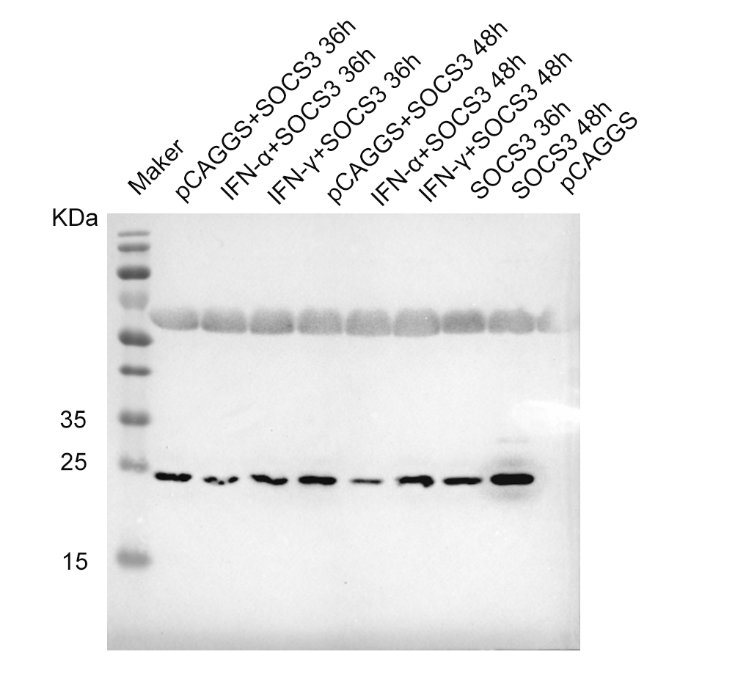

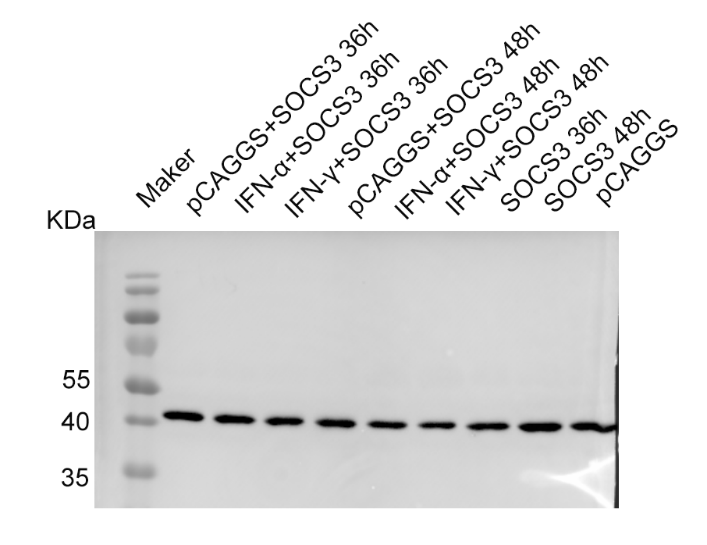


**Supplementary Figure 1.** Raw data of western blot. Interaction between IFNs and SOCS3. pCAGGS-SOCS3-His and pCAGGS-IFNα/γ-FLAG were co-transfected into CEFs, which were harvested 36 h and 48 h after transfection. Last three lines were the confirmation the expression of SOCS3 protein. Graph above is Western blot analysis of SOCS3 expression. Graph below is Western blot analysis of β-actin expression.

A


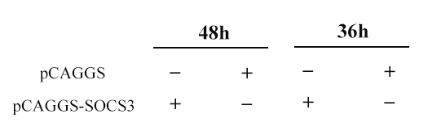

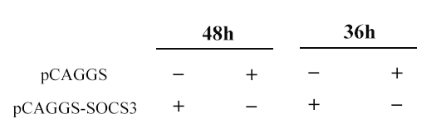


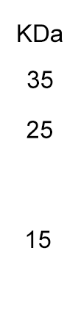


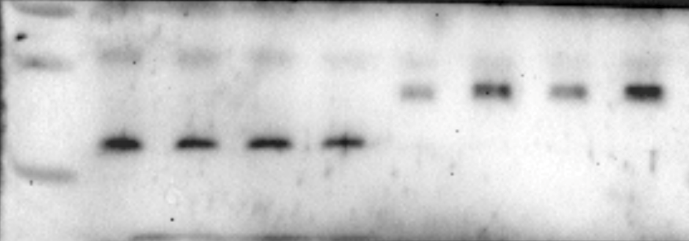


IFNα

IFNγ


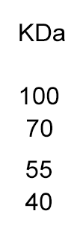

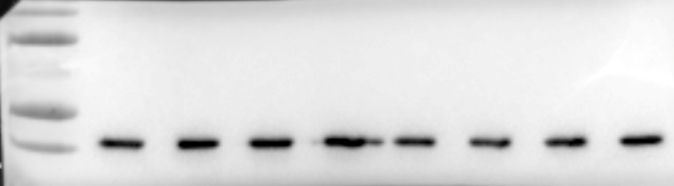
B


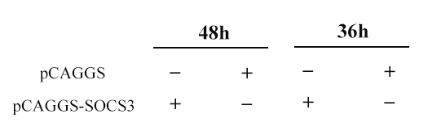

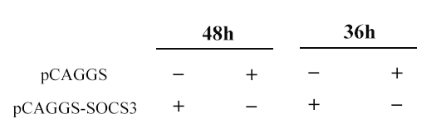


β-actin


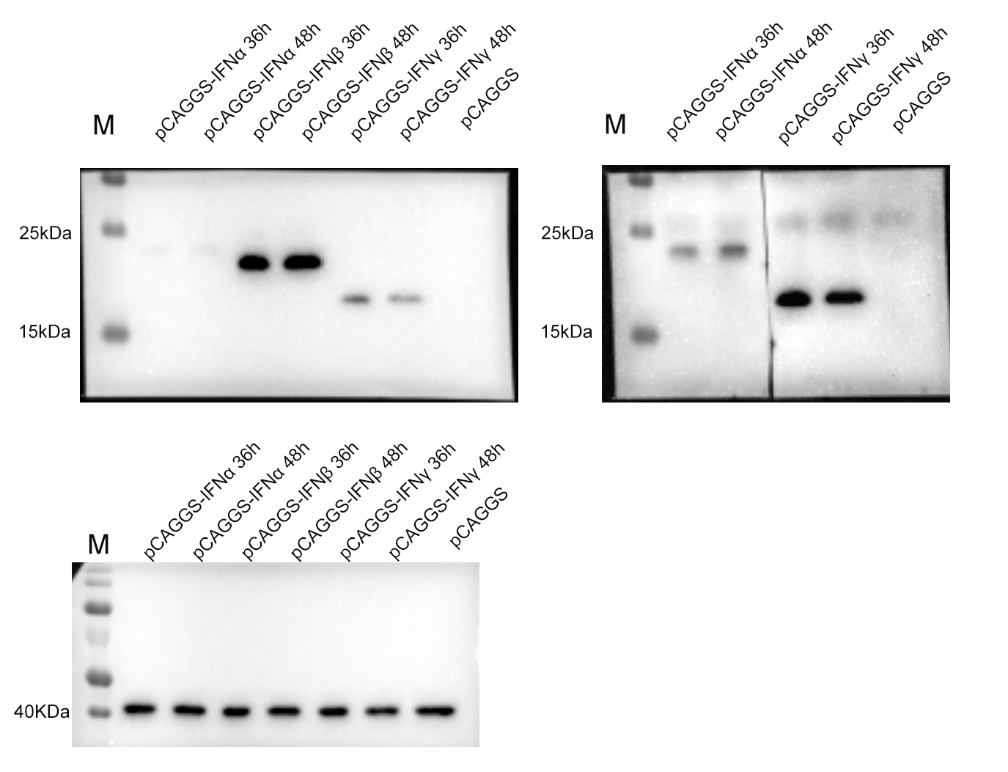

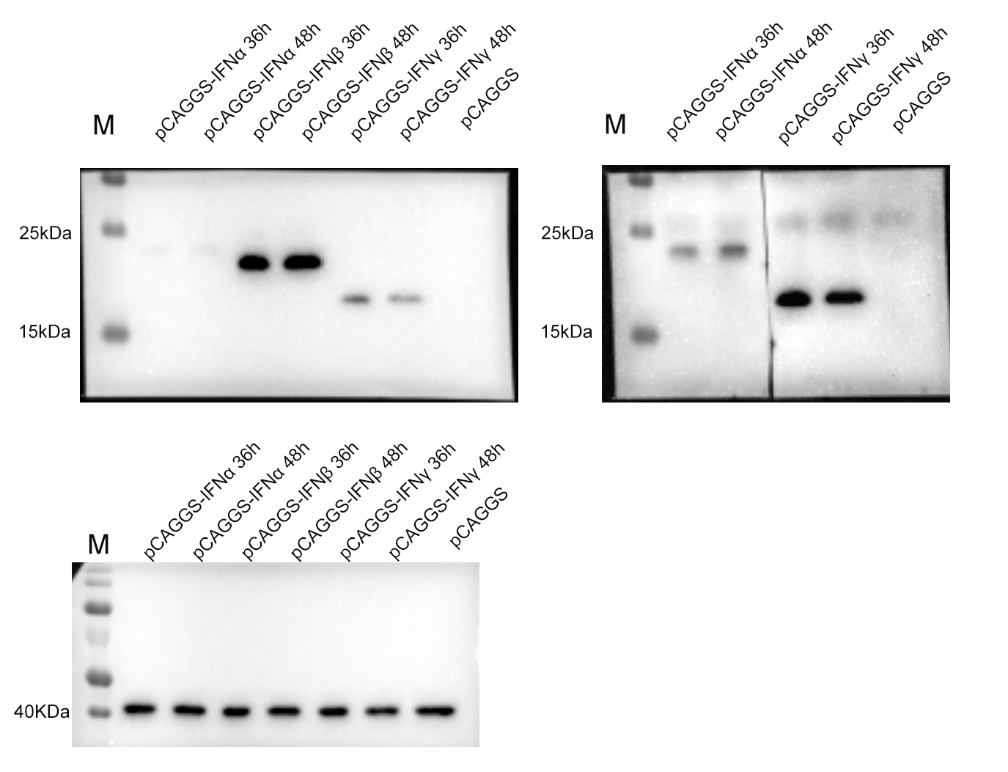
C

**Supplementary Figure 2.** Raw data of western blot. Interaction between IFNs and SOCS3. pCAGGS-SOCS3-His and pCAGGS-IFNα/γ-FLAG were co-transfected into CEFs, which were harvested 36 h and 48 h after transfection. (A) Western blot analysis of IFNα/γ-FLAG expression. (B) Western blot analysis of β-actin expression. (C) the confirmation the expression of IFNα/β/γ protein.


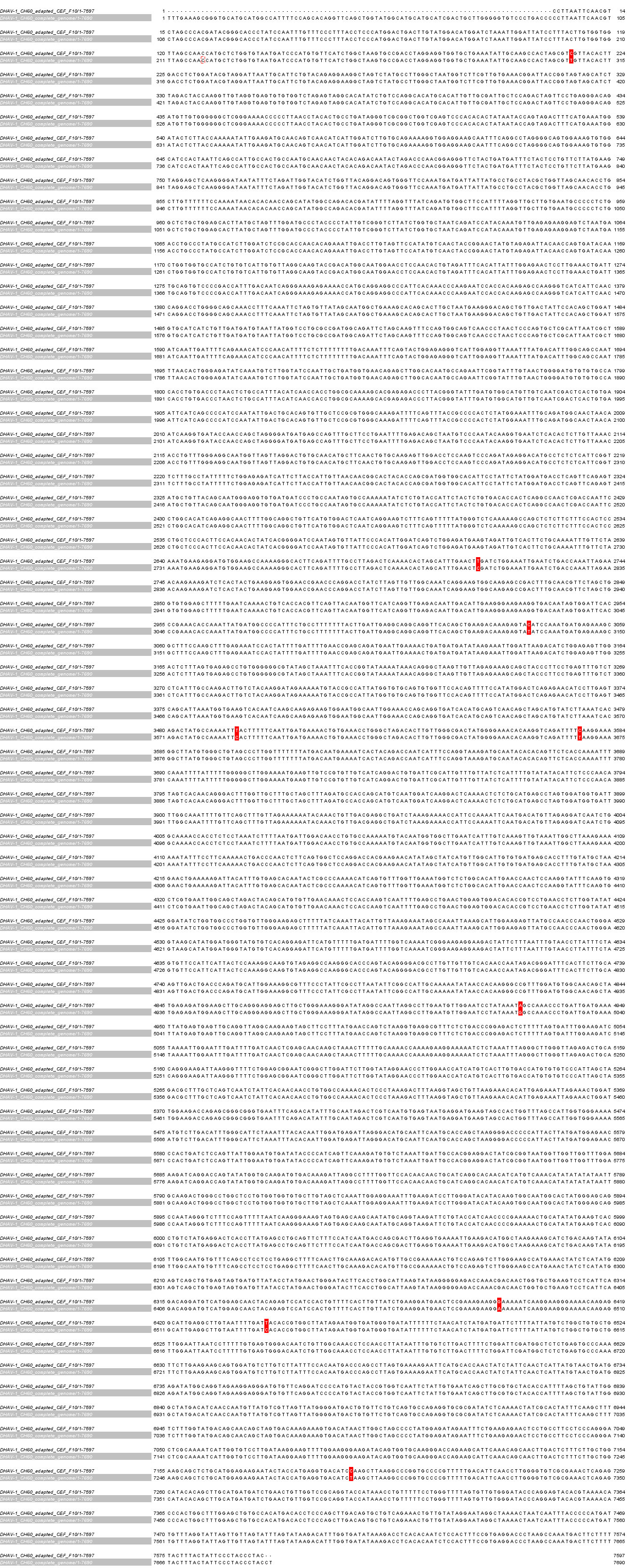


**Supplementary Figure 3.** The amino-acid sequence analysis of DHAV-1 CH60 strain and DHAV-1 CH60 adapted strain. red region indicates differential sequence.

A B


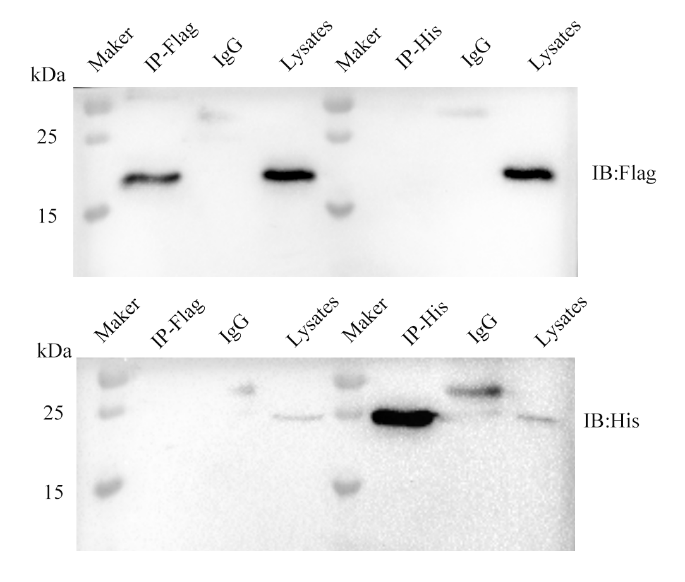

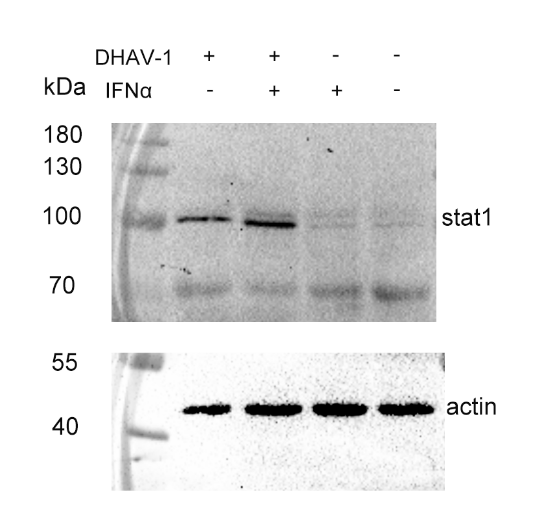


**Supplementary Figure 4.** Raw data of western blot. (A) Interaction of IFNα and SOCS3. CEFs were transfected with pCAGGS-IFNα-Flag and pCAGGS-SOCS3-His for 48 h before the coimmunoprecipitation and immunoblot analysis with the indicated antibodies. (B) Western blot analysis of STAT1 expression. DHAV-1 infected CEFs at 48 hpi and after the addition of 100 pg IFNα to stimulate CEFs at 37℃ for 15 min. Cells were harvested after stimulation.
